# Ultrasonic-driven adaptive control of robotic plasma arc cutting for bevel applications

**DOI:** 10.1007/s00170-025-15331-2

**Published:** 2025-03-18

**Authors:** Aasim Mohamed, Charalampos Loukas, Momchil Vasilev, Nina Sweeney, Gordon Dobie, Charles Macleod

**Affiliations:** Electronic & Electrical Engineering, University of Strathclyde, Royal College Building, 204 George St, Glasgow, G1 1XW Scotland, UK

**Keywords:** Automated bevel, Thickness measurement, Adaptive path generation, Parameters adjustment, Y-groove design, Robotic cutting

## Abstract

In heavy industries like oil and gas, and shipbuilding, maintaining process quality is challenging. These sectors face inconsistent manual procedures and a shortage of skilled operators regarding thermal cutting and bevelling for welding preparation tasks. Manual fitting and repetitive quality control modifications, especially during thermal cutting, significantly increase time consumption and hinder productivity. Traditional thermal cutting methods are prone to human error, resulting in inconsistent cut quality, and demand high expertise leading to variability in cut precision, increased rework, and material wastage. The objective of this work is to address these challenges by introducing real-time ultrasonic sensing into a robotic plasma cutting control system to automate the steel plate bevelling process. The ultrasonic sensor enables the system to dynamically adapt to variations in steel plate thickness before cutting, ensuring precise and consistent results. The solution begins by presenting an automated method for measuring thickness and computing bevel distance per sample. Secondly, it proposes adaptive adjustments to cutting parameters per sample, leveraging the ultrasonic sensor data to enhance accuracy and reduce the need for manual intervention. Finally, the approach introduces adaptive robotic path generation for cutting and utilizing real-time ultrasonic sensor data to optimize cutting paths. The outcome of this study is the successful development and validation of an adaptive robotic plasma cutting system for steel plate bevel applications, which leverages real-time ultrasonic sensor data to automate the parameter input process and robotic motion planning, demonstrating improved accuracy and efficiency compared to traditional approaches. The results demonstrate that ultrasonic-driven robotic cutting significantly reduces the average error cut percentage to 4.47% with deviations ranging from 0.13 to 0.23° for the bevel angle and 14.27% with deviations between 0.02 and 0.05 mm for root face deviation, compared to the standard cutting approach which has an average error of 18% with deviations ranging from 0.10 to 0.38 mm and 77.1% with deviation between 0.48 to 0.90°, respectively. This paper highlights the benefits of using advanced sensing technology, particularly ultrasonic sensors, to automate plasma bevel cutting for metal plates in the steel fabrication and welding sectors.

## Introduction

In recent years, plasma arc cutting (PAC) has emerged as a crucial technology for enhancing precision and production throughput in multiple heavy manufacturing sectors, such as oil and gas, nuclear, and steel fabrication. Plasma cutting systems have gained significant popularity in the industry due to their versatility and cost-effectiveness [1]. It can handle various forms of materials, including pipes, plates, rods, beams, and expanded sheets, with high tolerance to their condition, rust, dirt, or paint. With a diverse range of consumables available, they facilitate drag and standoff cutting, enabling access to confined areas and ensuring precise cuts on complex-shaped samples. This capability guarantees narrow, high-quality cuts on materials of varying thicknesses ranging from 0.50 to 130.00 mm depending on the plasma machine power capabilities [2]. Also, it is widely valued in the marine shipbuilding sector for its versatility and operational efficiency. Shipbuilders benefit from its capability to cut through various metal thicknesses, aligning with the diverse metal steel requirements; it influences fabrication times, repair, and maintenance procedures. This cutting method exhibits superior cutting speed on steels below 25.00 mm thickness compared to other thermal techniques such as oxyfuel cutting [3]. Metal fabricators across these various industries strive to meet the high supply demand along with quality requirements, while facing the challenges of an ageing skilled workforce and retirement. Process automation offers a valuable solution, optimizing operations and ensuring competitiveness. Many concerns related to the manual and semi-automated processes are still predominant in the industry, particularly in the fabrication of complex shapes. Manual fitting along with quality control verification accounts for over 50% of the cutting process time with only 20% attributable to the actual cutting [4].

**Fig. 1:**
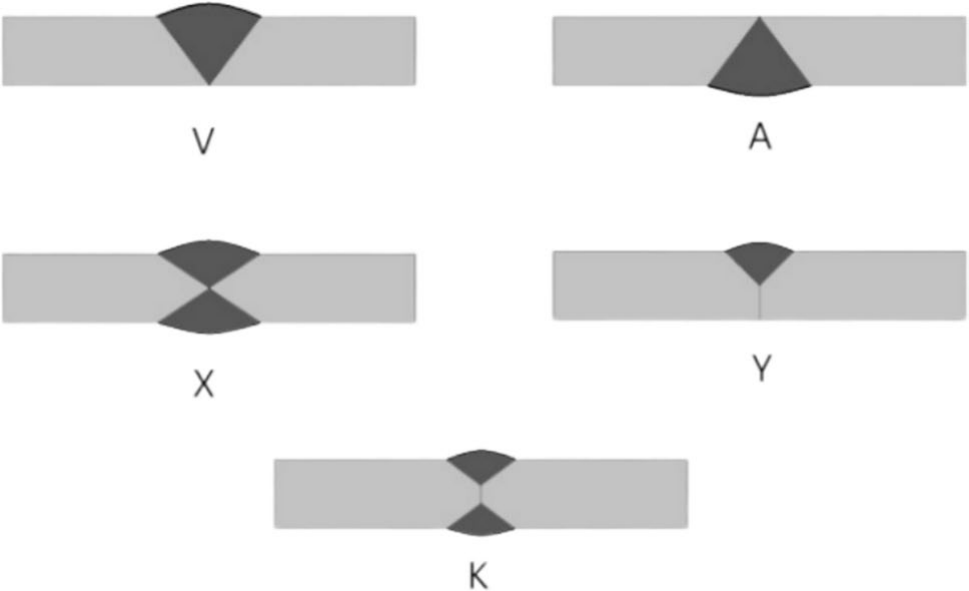
Joints type

Additionally, plasma cutting and bevelling are inextricably linked to welding and joining, a field dedicated to the process of joining pieces of material in various configurations [5]. There are five primary welding joint configurations: butt, lap, tee, edge, and corner joint types. The butt joint type includes several configurations, such as V, A, X, Y, and K as shown in Fig. 1. The complexity, cost, and quality of the required weld is determined by the joint’s design. The accuracy of weld joint bevelling significantly influences the weld quality [6], especially when conducted by automated welding systems which have emerged as transformative advancements within the welding industry, accelerating the joining of metals [7].

The recent advancements in manufacturing technologies like robotic manipulators enable manufacturers to produce complex parts and products with high accuracy and within a short time. Some examples include cutting steel pressure vessel parts, girders for steel fabricators, steel rods, pipework in shipyards, steel carriers for high-speed shuttle machines, and parts for high-pressure compressors [8]. Robotic manipulators are replacing the labour-intensive manual procedures in manufacturing processes. These systems have industrial robots integrated with sensors to perform cutting and welding tasks in an automated way, thus improving efficiency and accuracy as well as production output. Despite the developments made in welding and cutting technologies, the majority of bevelling processes are still performed by either manual or semi-automated methods [9]. Although these advancements have revolutionized different manufacturing industries, the use of robotics for steel plate bevelling remains limited. Key drivers of plasma automation include expedited production timelines, required precision for joining and fabrication applications, and minimizing the use of dangerous gases [10].

Ensuring that the cutting process maintains tight tolerance is crucial. Inaccurate cuts, wrong dimensions, and poor quality of cuts can lead to process challenges or unoptimized welding. This results in cumbersome secondary processes like grinding or even material replacement or scrap parts. There has been an increased interest in the automation and optimization of cutting processes [11]. To accommodate the research gap in bevelling automation for high production throughput that is required in heavy manufacturing sectors, this work presents a novel system for automated metal plate bevelling for cutting applications based on the actual thickness of the measured plate. The outcome is achieved through the integration of a robotic plasma cutting system with an ultrasonic thickness measurement sensor, equipped with real-time sensing capabilities to enable intelligent automation of the bevelling process. The proposed system can adapt and execute precise and repeatable cuts, resulting in shorter cycle times, boosting throughput, reducing the risk associated with human errors, and accommodating varying bevel angles and root faces. The novel elements of this research are described as follows:

1. Automated thickness measurement using real-time ultrasonic sensing.
2. Automated bevel distance calculation based on ultrasonic thickness measurements.
3. Adaptive per sample cutting parameters adjustment using ultrasonic measured thickness.
4. Adaptive robotic path generation for cutting, based on parameters determined above.

This paper is structured as follows: An introduction in Section 1. Section 2 provides a literature review of the optimization processes for plasma cutting parameters, focusing on their application in developing a robotic plasma cutting system for the steel plate bevelling process. It also identifies the research gaps within the state-of-the-art. Section 3 details the problem statement, framework, and algorithms for the adaptive robotic plasma bevel cutting concept. The results are analyzed in Section 4, and a managerial implication is in Section 5. The conclusion is presented in Section 6.

## Literature review

**Fig. 2:**
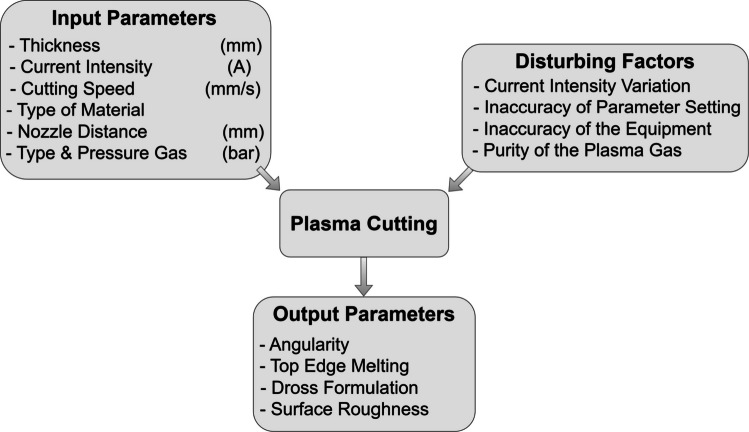
Process parameters

A comprehensive understanding of the plasma arc cutting process is crucial to achieve desirable outcomes. This entails precise knowledge of the involved parameters and their relevant effects as illustrated in Fig. 2. Input parameters refer to those variables that can be managed by a human operator. On the other hand, output parameters are linked to the quality of the resulting surface. Disturbing factors arise from the machinery, fitting, and working environment [12].

Some studies cover the topic of optimization of the cutting parameters in plasma arc cutting process. Authors in [13] studied the effect of process parameters on the dimensional accuracy of plasma arc cutting of a 10-mm stainless steel plate and presented an optimization study using the Taguchi L16 orthogonal array method and analysis of variance. Four process parameters were selected: arc voltage, current, standoff distance, and cutting speed. Each parameter varied across two levels. The standoff distance was found to be the most significant parameter, followed by current, and cutting speed. In [14], the authors conducted an optimization study of the plasma cutting process parameters using grey relational analysis. The experiments were designed using the Taguchi L9 orthogonal array, with stainless steel plates of 5 mm thickness being cut at different cutting speeds and plasma gas pressures while keeping other parameters constant. The quality of the cut was evaluated based on surface roughness, cut perpendicularity, and kerf width. Speed was identified as the most significant parameter. Authors in [15] investigated the effects of plasma arc cutting process parameters on the heat-affected zone and bevel angle in low-carbon steel. The experimental results were analyzed using response surface methodology, and a statistical model was developed to predict optimal cutting conditions. The study determined that cutting speed and arc current were the major parameters influencing the heat-affected zone, while torch-to-work distance significantly impacted the bevel angle.

In [16], the authors’ investigation focused on the impact of speed, current, voltage, and torch height on the quality of cut and material removal rate for stainless steel samples. The study aimed to optimize the PAC process using a combination of response surface method (RSM), grey relational analysis (GRA), and principal component analysis (PCA) as multi-objective criteria. They found that torch height, as well as the interaction between torch height and speed, was the most influential parameter in the PAC process. In [17], the authors investigated the ability to use a plasma arc for cutting simultaneously two parallel thin layers at different gap distances through the utilization of the Taguchi method and analysis of variance (ANOVA); optimal parameters (cutting speed, pressure, intensity, and gap size) were identified to minimize surface deformation. The optimization of the plasma arc cutting process identifies the optimal combination of parameters, settings, or variables, streamlining the process and minimizing waste. These approaches provide a structured framework for optimization and help in understanding the relationships between plasma cutting parameter variables. Also, the employment of statistical analyses helped in identifying significant parameters and determining the optimal settings based on objective criteria. On the other hand, the generalization of these settings to different materials, thicknesses, or specific requirements may vary. Implementing the proposed methods in practice may require further adjustments and validation based on specific conditions and constraints. Additionally, human intervention is required to implement the adjustments and reliance on accurate and reliable data for model development may pose challenges in real-world settings.

Researchers took a step ahead of traditional statistical techniques by employing artificial intelligence and modelling techniques for the optimization of the PAC process. In [18], the authors demonstrate the use of a three-layer feed-forward backpropagation artificial neural network to model and predict surface roughness, considering the cutting parameters. The experiments involved working with 15-mm-thick structural steel plates where the cutting current and speed were varied as input process parameters. The same authors in [19] built a similar ANN model but introduced cutting height as an additional input process parameter for modelling the kerf width in plasma jet cutting. Both models exhibited satisfactory performance as they successfully predicted surface roughness and curve width values based on the input process parameters. Authors in [20] combined an ANN model with a neuro-fuzzy model called an adaptive neuro-fuzzy inference system (ANFIS) for exploring the impact of cutting current, cutting speed, and material thickness on the prediction of kerf width. ANFIS combines the learning ability of ANN with the knowledge representation capability of fuzzy logic systems. ANN and ANFIS models proved their ability to capture complex patterns, adapt to new data, and provide accurate predictions for the plasma cutting process. The employment of AI and modelling techniques in optimizing the plasma arc cutting process offers several advantages including minimizing cycle times by expediting the parameter optimization process, and the need for manual trial and error. Predicting process performance and outcomes based on historical data and real-time inputs. On the other hand, there are some disadvantages to consider. The complexity of AI models and the limitation of interpretability and transparency. The need for large and representative datasets, which can be time-consuming and resource intensive, in addition to the need for a highly skilled workforce.

A limited number of scientific studies have addressed the use of robotic plasma bevelling systems for steel plates. Most of the studies found in the literature discuss the pipe-cutting applications. Most of the progress is made by industries and patents. However, authors in [21] presented the development and evaluation of a novel plasma cutting approach for efficiently producing a Y-groove shape. The key methodological innovation is designing a specialized plasma Y-groove cutting machine that employs a three-dimensional link-type torch block to tilt the plasma torch. This allows for faster, more compact, and durable cutting head movements. The cutting process involves two passes. The main advantages of this integrated plasma Y-groove cutting approach include increased accuracy in cutting, a decrease in the overall time needed for the process, and the removal of the need for machines that are specifically used for groove cutting. However, the paper also points out some potential drawbacks as well, such as it is less effective for small parts where specific machines used for groove cutting may be more appropriate. Also, the two-stage cutting of the Y-groove shape has technical challenges in terms of maintaining dimensional accuracy with root face deviations from 0 to + 0.5 mm and a bevel angle deviation of ± 1.5°. The approach still relies on the operator for setting the cutting parameters, sample thickness, and path planning. In [22], the authors presented a five degrees of freedom gantry robot which is used to automate the cutting of Y-shaped welding grooves of a steel plate. The system incorporates flame cutting as the primary method of cutting with the help of vision-based edge detection and laser range finders (LRFs) for measuring the thickness of the plate edges. The study assumes a fixed plate thickness using the (LRFs) reading and neglects natural variations in material thickness tolerances. Also, the study did not address the need for an adaptive approach to adjust the cutting parameters based on the actual plate thickness. This can lead to errors in cutting parameter selection, tool positioning, and trajectory planning, ultimately affecting the quality of the cuts. Furthermore, while vision and laser systems enable automation, they can be less reliable than direct thickness measurements using ultrasound sensors, as surface irregularities and environmental factors may impact the accuracy of the measurements.

### Research gap

The previous studies highlight the critical importance of considering how material thickness impacts the plasma arc cutting process, particularly in relation to the cutting parameters and output quality. Variations in material thickness and plate flatness, which are significant factors used to determine cutting parameters such as cutting current, speed, torch-to-work distance, and gas pressure, can lead to errors in the final bevel cut dimensions. Therefore, it is essential to manage these thickness variations due to their direct impact on the accuracy of cuts, alignment, and the overall structural integrity of the assembled parts. To mitigate the risk of dimensional inaccuracies, a careful monitoring and control process of sample thickness and cutting parameters is necessary to ensure that the final components meet the required accuracy standards.

The research gap identified by previous studies indicates the following:

1. Lack of an automated method for identifying optimal cutting parameters based on measured actual plate thickness.
2. Absence of automated robotic path planning based on actual plate thickness.
3. Overreliance on trial and error.

Table 1 illustrates the contributions to novelty achieved in the current work by introducing the adaptive plasma arc cutting system for steel plate bevel applications.

**Table 1:** The progress in optimizing the plasma cutting parameters and adaptive cutting approaches

Relevant work	Adaptive input parameters setting	Adaptive bevel distance calculation	Adaptive path planning
[13–17]	✗	✗	✗
[18–20]	✓	✗	✗
[21]	✗	✗	✓
[22]	✗	✓	✓
This work	✓	✓	✓

## Problem statement

As an illustrative example of the importance of thickness control, we can refer to the European Standard EN 10029, which provides precise guidelines for acceptable thickness tolerances in hot-rolled steel plates made of both non-alloy and alloy steel with a nominal thickness of 3.00 mm or more. For example, when considering plates with a thickness of 8.00 to 15.00 mm, common in heavy industry applications, the plate thickness tolerance is − 0.85 to + 0.85 [23]. These thickness tolerances standard play a crucial role in the plasma cutting process, as variations in plate thickness can significantly affect the accuracy and quality of the resulting cuts.

### Key challenges

- Ensuring consistent bevel angle and cutting depth during operation.
- Detecting real-time thickness to adjust the cutting speed accordingly.
- Optimizing operation time while maintaining high-quality cuts.

### Visual representation of the problem

**Fig. 3:**
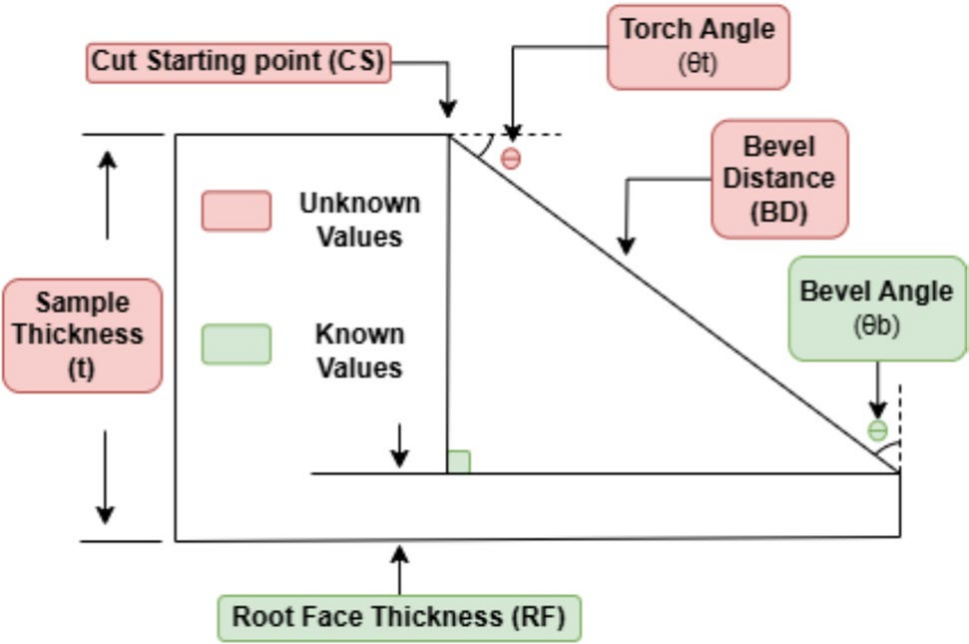
Y-groove design parameters

The design requirements and parameters for the (Y) groove joint consist of key factors such as the thickness of the part (*t*), bevel cut distance (BD), the angle of the bevel (${\theta}_{b}$), the size of the root face ($\text{RF}$), cutting starting point ($\text{CS}$), torch angle ${(\theta }_{t})$, and cutting speed (ES) as shown in Fig. 3. These parameters are essential for the Y-grove cutting design process. These parameters must be determined and calculated individually for each sample. This added complexity in setup and programming, increases the likelihood of human-related errors and extends the processing time, ultimately compromising the precision of the bevel angle, root face, and cutting depth.

Table 2 summarizes the key notations and their definitions, providing a reference for understanding the parameters involved in the Y-groove cutting design process.

**Table 2:** Parameter definitions and notations

Notation	Definition
CS	Cut starting point – The initial point where the cutting process begins on the sample
$\theta_b$	The angle formed between the surface of the material and the edge of the cut
BD	Bevel distance – The distance defining the separation or offset of the bevel edges
RF	The flat side at the bottom of the bevel that remains uncut
$\theta_t$	The angle at which the cutting torch is oriented relative to the workpiece
***t***	The thickness of the material being cut
ES	Cutting speed

### Proposed mathematical model

When dealing with a right triangle provided with a known angle (bevel angle) and side length (sample thickness), the process of determining the remaining measurements involves the use of trigonometric principles. First, the known angle is identified, and its relationship to the sides of the triangle is established. Next, the selected trigonometric ratio is utilized to compute the value of the unidentified side. By utilizing the bevel angle and sample thickness, the unknown measurements can be determined with the help of algebraic manipulation. It is crucial to exercise caution in selecting the appropriate trigonometric function, ensuring that it aligns with the relationship between the known side and angle [24]. Through the implementation of trigonometric principles and a systematic approach, the situation of having one known side and angle values in a right triangle can be resolved, yielding a comprehensive comprehension of its geometry and measurements.

Ultrasound technology is employed to meet the requirement of acquiring information of a single-sided measurement by determining the thickness (*t*) of the sample. User input is necessary to enter the desired bevel angle (${\theta }_{b}$) and root face size (RF), which satisfy the condition of obtaining the angle value. In this application, the thickness measurement corresponds to the value of the adjacent side, while the bevel angle represents the second angle value, and the first angle is 90°. The desired root face is subtracted from the measured thickness. The values of the remaining measurements (hypotenuse and opposite side) and the torch angle can be calculated using the following:

#### Bevel distance

The importance of this calculated measurement is in determining the desired cutting length (bevel distance), which is crucial for obtaining precise readings to effectively control and adjust the cutting speed.

1 $$BD=\frac{t\, -R\, F}{\text{sin}({90}^{^\circ }-\,{\theta }_{b})}$$

#### Cut starting point

The calculated measurement represents the initial position of the cutting path in relation to the plate’s edge in x direction. The plasma kerf width, which is identified as the width of the cut [25], needs to be taken into consideration during path planning. The required root face determines the desired torch-to-work distance. This method was able to perform the cut and address the roof face within a single cutting path.

2 $$CS=\frac{t\, -R\, F}{\text{cos}({90}^{^\circ }-\,{\theta }_{b})}$$

#### Torch angle

In accordance with the principles of right-angled triangles, the first known angle is fixed at 90°, which serves as the initial measurement. The user provides the second measurement, which is the bevel angle. By subtracting the required bevel angle from 90°, the torch angle can be calculated.

3 $${\theta }_{t}={90}^{^\circ }-\boldsymbol{ }{\theta }_{b}$$

### Proposed automated bevel cutting system

**Fig. 4:**
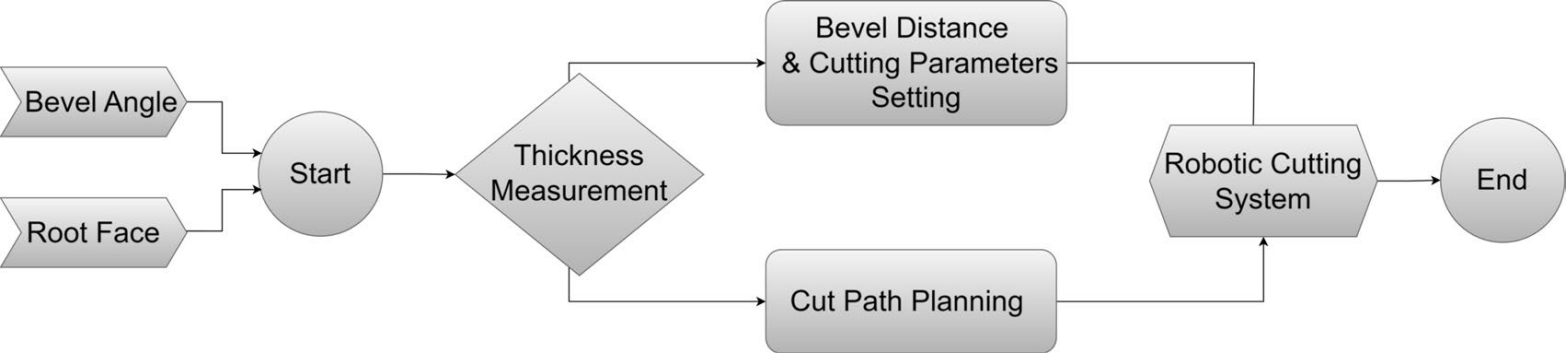
Process flow chart

The flow chart in Fig. 4 describes the proposed process utilized by a robotic cutting system and ultrasonic sensor, beginning with predefined input parameters (Root Face) and (Bevel Angle) set by the user. These parameters establish the initial conditions for the adaptive cutting approach. Then, the process starts with an automatic thickness measurement for each sample, accurately determining its thickness. This critical step ensures that the bevel distance and remaining cutting parameters, such as bevel distance (BD) and tool path starting point (CS), are precisely adjusted according to the specific thickness of each sample.

**Fig. 5:**
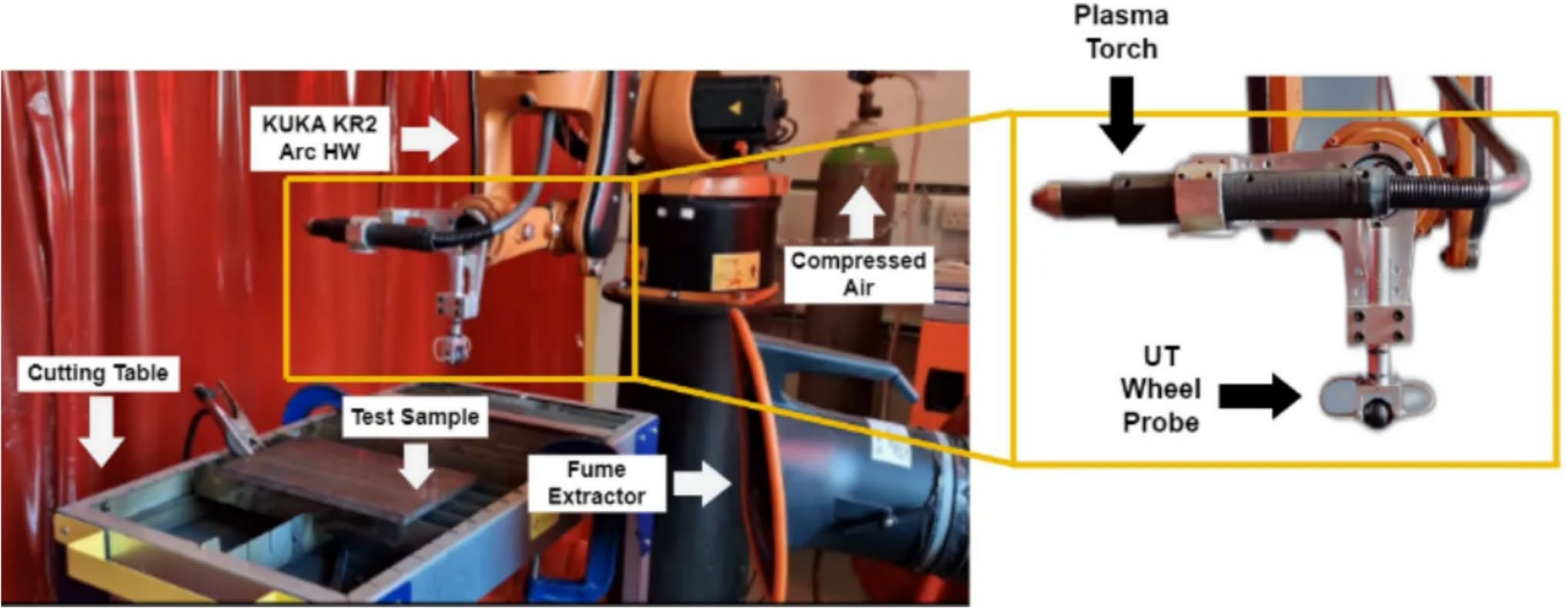
Adaptive robotic plasma cutting system

**Fig. 6:**
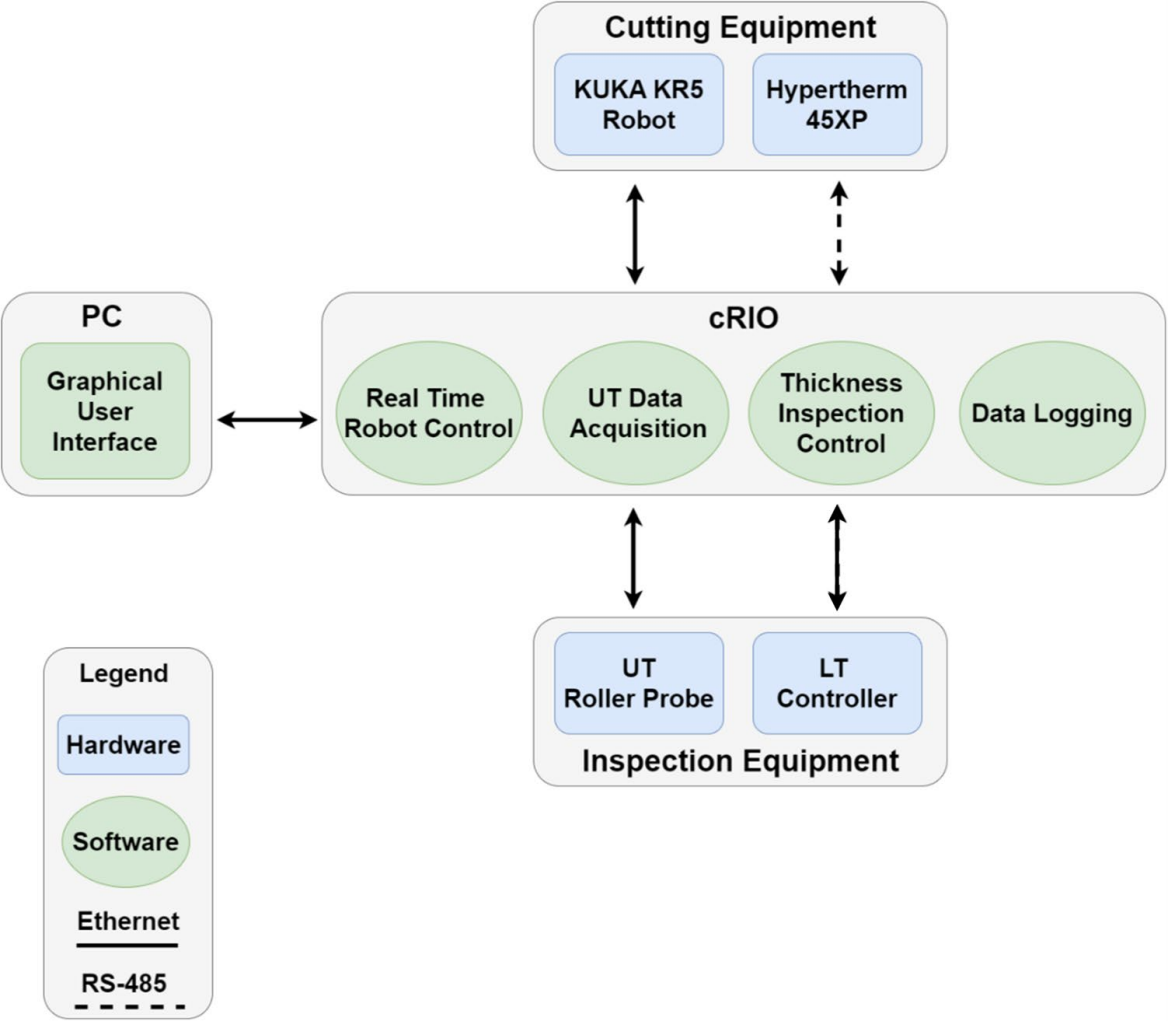
System architecture

Following this, the “Cut Path Planning” phase assigns the cutting starting point for trajectory planning which the tool will follow in a straight line with the required torch tilt angle. This is crucial for achieving the required precision and meeting the specifications of the cuts. The robotic cutting system then executes the cutting, adhering to the planned path and adjusted parameters. The process concludes at the “End” stage, marking the completion of the cutting for each sample. The proposed system illustrated in Fig. 5 consists of the following parts:

A KUKA KR5 Arc HW, a 6-degree-of-freedom industrial manipulator [26] deploys a customized aluminium end-effector accommodating the cutting torch and ultrasonic probe. The ultrasonic probe is a 5 MHz split crystal dry-coupled wheel probe from Eddyfi Technologies. This probe is capable of measuring thicknesses ranging from 2.50 up to 100.00 mm [27], and it is controlled by a PEAK LT ultrasonic controller [28]. The Hypertherm Powermax 45XP plasma enables the cutting of steel specimens up to 25.00 mm in thickness [29]. The overall system is controlled by a National Instruments CompactRIO (cRIO) 9038 controller, while a host PC deploys a custom graphical user interface (GUI). Figure 6 provides a high-level diagram illustrating the system architecture.

### Ultrasonic thickness measurement

Ultrasonic thickness gauging is a widely employed method to measure the thickness of various materials. This technique utilizes sound energy at frequencies beyond human hearing, typically between 500 kHz and 20 MHz, and it is commonly based on the time-of-flight for an ultrasonic wave which is generated by a transducer to travel through the material. The following formula for thickness measurement is given by (4).

4 $$T=V \frac{t}{2}$$

where *T* is the thickness, *V* is the velocity of sound in the material, and *t* is the measured round-trip transit time. It is crucial to calibrate the gauge to the specific material being measured for accuracy. The choice of transducer depends on factors such as the material type, thickness range, geometry, temperature, and accuracy requirements. Ultrasonic thickness gauging is suitable for various materials, including metals, plastics, composites, and ceramics, making it a versatile method for thickness measurement [30]. In the context of ultrasonic thickness measurement, various transducer types are available, each tailored to specific applications. Contact transducers, characterized by their simplicity, are widely favoured by technicians due to their direct contact with the test sample. In contrast, delay line transducers excel in the measurement of thin materials by utilizing a cylindrical delay line to segregate external echoes from the primary pulse emitted by the test material. Immersion transducers are particularly effective for dynamic measurements and use a water column to transmit sound waves into the test material. Conversely, dual-element transducers find their niche in scenarios involving rough, corroded surfaces, and are exclusively employed with corrosion gauges [31].

**Fig. 7:**
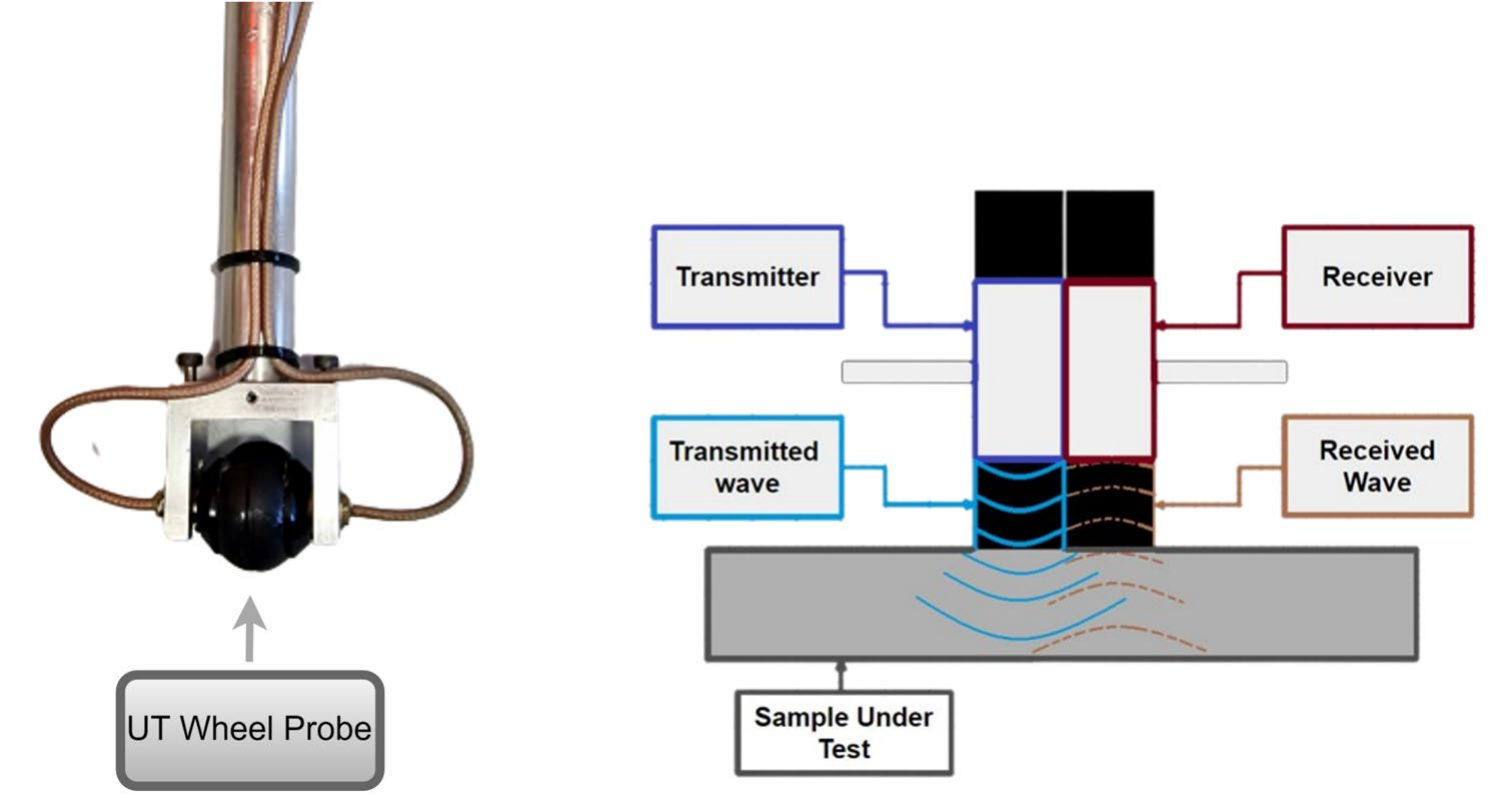
Ultrasonic sensor internal diagram

In this study, an ultrasonic wheel probe is used which is a specialized device that combines a wheel or roller mechanism with ultrasonic transducers to conduct measurements on various surfaces under harsh environments [32]. The design of the used wheel probe includes two rotating solid rubber tyres on the sensor base. This unique design allows the probe to acquire signals without the need for surface liquid coupling. It enables the transmission and reception of ultrasonic signals into and from the specimen. The dual-element transducers as shown in Fig. 7 have two separate parts in one unit. The transmitter emits ultrasonic signals, while the receiver captures any reflected signals. The two piezoelectric elements of the transducer are positioned at an angle toward each other, creating a sound path that intersects with the tested material. This design makes them well-suited for tasks such as thickness measurement [33].

### Signal processing

**Fig. 8:**
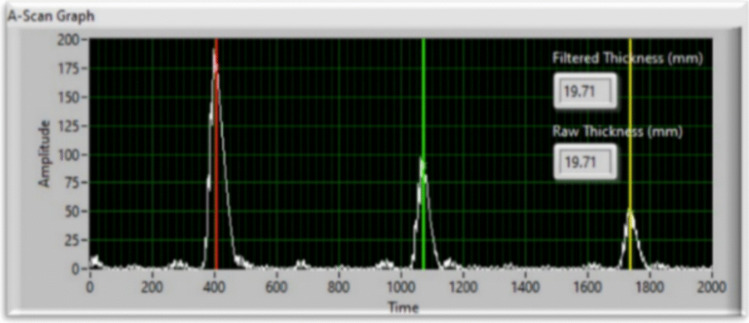
Ultrasonic A-scan signals

There are some challenges related to the compressive nature of the wheel probe tyre and the discrepancy in sound propagation velocity between the tyre and the underlying material can affect the acquisition of accurate readings. To overcome these limitations, the thickness of the base material was determined using a method that involved averaging three or more successive back-wall echoes. Various factors can introduce variations in the coupling between the tyre and the surface. These factors include reduced pressure of tyre-to-surface contact, variations in the height of the robot end-effector relative to the surface, and localized surface irregularities. These variations in coupling result in corresponding changes in the amplitudes of multiple back-wall echoes. Delayed time gating is used to ignore early arriving echoes from the probe tyre and focus exclusively on echoes from deeper within the material. This allows for a clearer assessment of internal echoes, leading to measuring the material thickness accurately. The largest peak observed within the recorded time window corresponds to the first back-wall echo which is the reflections from the material’s opposite side, while the subsequent peaks represent internal reflections of the ultrasonic wave as shown in Fig. 8. By considering three back-wall echoes, the thickness of the tested material can be calculated using the following [34].

5 $${S}_{t}=\frac{{V}_{c}* ({t}_{3}-{t}_{1})}{4}$$

The calculation of sample thickness ${S}_{t}$ can be determined using the speed of sound in the material ${V}_{c}$ and the time indices of the first back-wall echo ${t}_{1}$ and the third back-wall echo ${t}_{3}$. The peak detection method used in this study demonstrates excellent sensitivity and robustness, effectively accommodating variations in the amplitudes of both the transmitted wave and the first, as well as subsequent echoes.

The process of thickness measurement was continuously updating the setpoints within the real-time motion control loop. The ultrasonic controller was set up to capture 8-bit rectified A-scans with a Pulse Repetition Frequency (PRF) of 20 kHz. To minimize electromagnetic noise emitted by the robotic manipulator, a 128-sample averaging technique and a bandpass filter ranging from 3 to 15 MHz were implemented. All received ultrasonic signals were processed and transmitted over ethernet to the cRIO controller for processing. Adaptive detection thresholding was employed to identify the peaks of back-wall reflections in the material, aiming to minimize the impact of coupling variations. The threshold value, determined as 30% of the largest detected peak amplitude, ensured capturing multiple successive echoes. Allowed for the utilization of three or more peaks to calculate the thickness.

### Adaptive cutting parameters adjustment

Using the manufacturer’s cutting charts [29] in conjunction with the proposed ultrasound sensor–based thickness measurement, the required cutting parameters can be estimated. In this method, the ultrasound sensor is employed to accurately measure the thickness of the material being cut. By referencing the specific thickness measurement obtained through the ultrasound sensor, the corresponding cutting parameters can be determined from the cut charts. This approach allows for a more precise and tailored selection of cutting parameters based on manufacturer recommendations, accounting for the actual material thickness rather than relying solely on estimations or generic settings. Ensuring optimal cutting parameters settings for a given material thickness.

Estimated cutting speed (ES)*:*

**Fig. 9:**
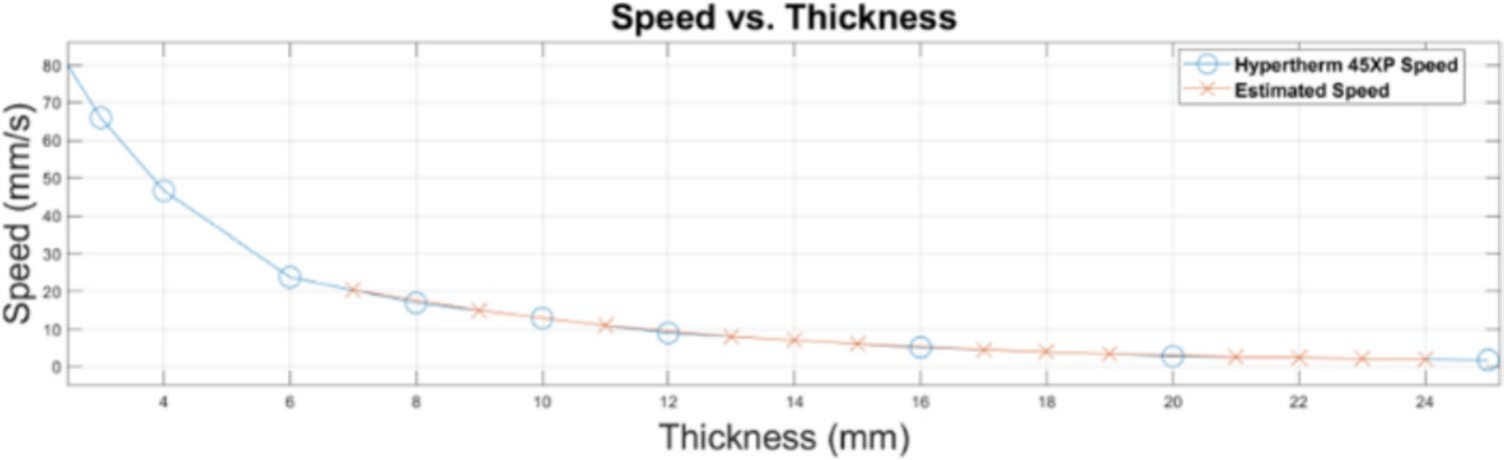
Cut speed vs thickness

To address the gaps and missing values in the dataset, particularly those related to cutting speeds at specific thicknesses, a recommended approach is employing linear interpolation [35]. This method assumes a linear relationship between material thickness and cutting speed within the known data points range. To estimate the cutting speed (ES) for a specified missing thickness (t), we first identify the nearest known thickness values $({t}_{1})$ and ${(t}_{2}$) that bracket (*t*). These known thickness values are essential reference points. Linear interpolation extrapolates linearly between the known cutting speeds at $({t}_{1})$ and $({t}_{2})$. The formula used for estimation is as follows [36]:

6 $$ES=\frac{\left(t-{t}_{1}\right)*(Speed\, at\, {t}_{2}-Speed\, at\, {t}_{1})}{{t}_{2}-{t}_{1}}$$

(*t*) represents the specified thickness for which we want to estimate the cutting speed. ${(t}_{1})$ and ${(t}_{2})$ are the nearest known thickness values that surround (*t*). Figure 9 illustrates the provided speed versus the estimated speed using this method. Linear interpolation offers a precise estimation method when there is a linear relationship between thickness and speed within the vicinity of known data points. It provides accurate speed predictions that bridge the gaps in the dataset. By applying this method, the cutting speeds for missing thickness can effectively be calculated, ensuring accurate and efficient cutting operations across a range of material thicknesses.

### Torch height control (THC)

Torch height control (THC) is an essential component of the mechanized cutting process, and it is responsible for automatically adjusting the distance between the torch and the workpiece during the cutting process to maintain a steady distance. This adjustment is achieved by monitoring and regulating the arc voltage. The current supplied by the plasma power source remains stable during cutting, while voltage fluctuates based on the separation between the torch’s electrode (cathode) and the material being cut (anode). According to Ohm’s law, voltage is directly proportional to resistance, and the resistance in the arc depends on the distance [37].

## Results

A total of eighteen steel samples divided into two groups, each with dimensions of 200 mm × 300 mm, were included in the experimental study. Each group possessed varying tolerated thicknesses. The input parameters for the bevelling process were the bevel angle (30°) and the root face (2.00 mm). The selection of a 30° bevel angle and a 2-mm root face is widely used in industries such as construction, shipbuilding, and pressure vessel fabrication due to its balance of accessibility, cost-efficiency, and mechanical integrity. These parameters are particularly suitable for materials with thicknesses ranging from 5 to 20 mm, offering optimal weld pool access, reduced filler metal usage, and controlled penetration to prevent burn-through. Recommended by ISO 9692–1 and British Standards, they are commonly applied in structural steel welding, pipelines, and high-pressure equipment [38]. Cutting parameters were obtained from the updated cut chart, to determine the optimal cutting parameters for each thickness. Plates with nominal thicknesses of 8.00 mm, 10.00 mm, and 15.00 mm, which are commonly used for joining purposes in the shipbuilding industry, pipelines, and other related heavy industries, were chosen [39]. These dimensions are significant in the context of the industries mentioned due to the specific requirements for strength, durability, and safety [40]. The samples were procured and then machines to match tolerance limits. Nine samples were subjected to the standard cutting approach, while the other nine were subjected to the adaptive cutting approach. For further details about the experimental samples, refer to Table 3.

**Table 3:** Samples specifications

Thickness tolerance (mm)	Samples thickness (mm)					
		Qty		Qty		Qty
− 0.85	7.15	2	9.15	2	14.15	2
0.00	8.00	2	10.00	2	15.00	2
+ 0.85	8.85	2	10.85	2	15.85	2

ISO 9013:2002 represents a globally recognized standard which outlines the specifications and acceptable tolerances for thermal cutting processes. The standard applies to plasma cuts ranging from 0.50 to 150.00 mm [41]. The surface quality is defined using specific parameters from the standard. Angularity tolerance, as well as root face consistency, taken into consideration in this study with the aim of targeting the accuracy of the bevel cut and the consistency of the root face. The concept of angularity tolerance signifies the deviation acceptable in the angular evaluation of a component or feature from a specified angle. It defines the extent to which the angle can deviate while still aligning with the defined quality. This tolerance holds importance in preserving precise angular positioning or relationships between components, ensuring proper operation, assembly, or performance within a defined spectrum of angular values.

### Input parameters

Table 4 illustrates the parameters used in the standard approach, which assumes a consistent bevel distance, cutting speed, and a fixed starting point for cutting, regardless of material thickness tolerance for each group. This approach does not take into account the variations in thickness that might affect the accuracy of the bevel cutting process.

**Table 4:** Standard approach input parameters

30 degree bevel – 2 mm root face				
Tolerance (mm)	*t *(mm)	CS (mm)	BD (mm)	ES (mm/s)
− 0.85	7.15	3.46	6.92	20.41
0.00	8.00	3.46	6.92	20.41
+ 0.85	8.85	3.46	6.92	20.41
− 0.85	9.15	4.61	9.23	15.00
0.00	10.00	4.61	9.23	15.00
+ 0.85	10.85	4.61	9.23	15.00
− 0.85	14.15	7.50	15.00	6.12
0.00	15.00	7.50	15.00	6.12
+ 0.85	15.85	7.50	15.00	6.12

Table 5 illustrates the parameters used in the experiments for the adaptive approach. The bevel distance and cutting starting point were calculated using (1) and (2), respectively, based on per sample thickness measurements and operator inputs (bevel angle – root face). Cutting speed was determined using (6), with reference to the Hypertherm cut chart.

**Table 5:** Adaptive approach input parameters

30 degree bevel – 2 mm root face				
Tolerance (mm)	*t *(mm)	CS (mm)	BD (mm)	ES (mm/s)
− 0.85	7.15	2.97	5.94	23.83
0.00	8.00	3.46	6.92	20.41
+ 0.85	8.85	3.95	7.90	17.00
− 0.85	9.15	4.12	8.25	17.00
0.00	10.00	4.61	9.23	15.00
+ 0.85	10.85	5.10	10.21	10.00
− 0.85	14.15	7.00	14.00	7.08
0.00	15.00	7.50	15.00	6.12
+ 0.85	15.85	7.99	15.99	5.10

As material thickness increases or decreases, the cutting starting point (CS) proportionally shifts higher or lower in X direction representing the start cutting point. The adaptive technique ensures the cutting path is maintained within the required cut path. This adjustment is critical to ensure the accuracy of the cutting path, meeting the desired cut specifications. Similarly, the bevel distance (BD) follows this trend, an increase in bevel depth is associated with an increase in thickness tolerance, and vice versa. The inverse relationship between estimated cut speed (ES) and material thickness (t) is crucial for maintaining accurate cutting speed as seen in the table above. Ensuring the consistency and effectiveness of the cutting parameters. The data underscores the precision and adaptability of the adaptive bevelling approach, autonomously calculating and adjusting these parameters for each thickness variation. This adaptability presents a significant advantage over traditional methods, which rely on manual adjustments that may lack the same level of automation. Notably, the system’s capacity to accommodate thickness tolerances of + 0.85 mm and − 0.85 mm is important as proof of concept. This versatility guarantees the accuracy of the cutting process, even when material thickness fluctuates within these tolerances.

### Cut quality

**Fig. 10:**
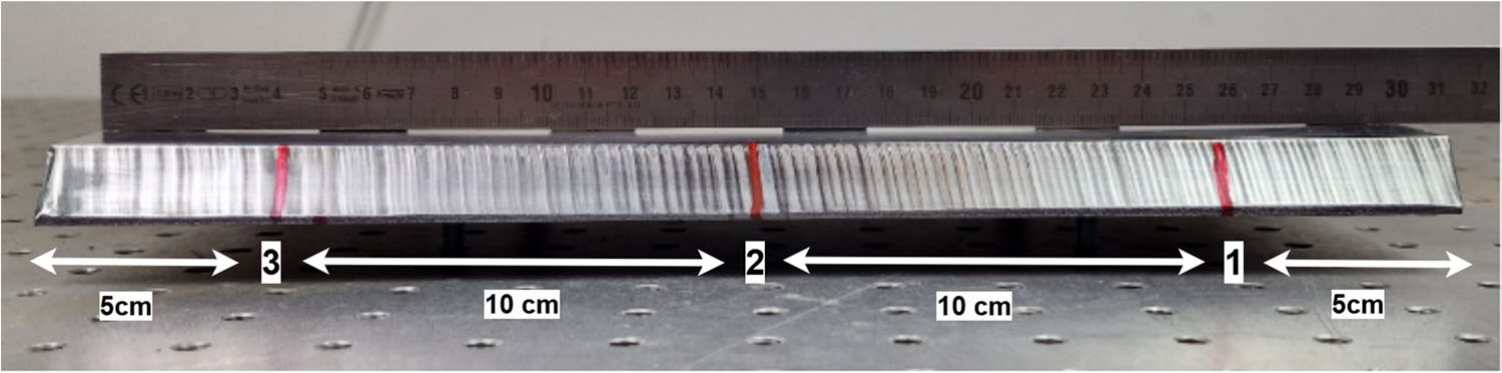
Measurement points

In the analysis of data related to standard cutting and adaptive cutting approaches and their cutting quality, the focus is on bevel angle deviation (degree) and root face deviation (mm). Figure 10 highlights deviation measurement points associated with these parameters for the experiment samples, providing valuable insights into cut quality. Three measurement points (1, 2, 3) were used to validate the bevel angle and root face diameter.

Table 6 illustrates the measured deviations of the standard approach samples. For bevel angle deviation, we observe a range of 0.48 to 0.90 deg as the material thickness tolerances vary from 7.15 to 15.85 mm. This indicates an increase in bevel angle deviation with greater thickness tolerance. Regarding root face deviation, standard cutting reveals deviations ranging from 0.10 to 0.38 mm as the material thickness tolerance increases. The high deviations relative to thickness tolerance groups indicate a direct impact of thickness variation on cutting input parameters, such as cutting speed (ES), cutting starting point (CS), and cutting distance (BD), resulting in lower dimensional accuracy of the output cut quality of the standard approach.

**Table 6:** Deviation values of bevel angle and root face of standard cutting approach

Standard cutting			
Tolerance (mm)	Thickness (mm)	Bevel angle deviation (degree)	Root face deviation (mm)
− 0.85	7.15	0.48	0.29
0.00	8.00	0.86	0.10
+ 0.85	8.85	0.78	0.31
− 0.85	9.15	0.90	0.15
0.00	10.00	0.62	0.25
+ 0.85	10.85	0.62	0.19
− 0.85	14.15	0.44	0.11
0.00	15.00	0.62	0.10
+ 0.85	15.85	0.73	0.38

In Table 7, the adaptive cutting approach displays a narrower deviation range of 0.13 to 0.23 deg across the same thicknesses for bevel angle. A different pattern emerges for the root face with deviations between 0.02 and 0.05 mm maintaining a significantly lower root face deviation, indicating improved adaptability of the system, regardless of material thickness tolerance. In the adaptive approach, cutting input parameters are updated dynamically based on the actual thickness measurements of the sample. This ensures better alignment between the cutting parameters and the sample’s actual thickness, compared to the uniform thickness assumption used in the standard approach. As a result, the adaptive cutting approach achieves improved dimensional accuracy, reduced deviations, and enhanced time efficiency.

**Table 7:** Deviation values of bevel angle and root face of adaptive cutting approach

Adaptive cutting			
Tolerance (mm)	Thickness (mm)	Bevel angle deviation (degree)	Root face deviation (mm)
− 0.85	7.15	0.21	0.06
0.00	8.00	0.18	0.02
+ 0.85	8.85	0.15	0.02
− 0.85	9.15	0.16	0.02
0.00	10.00	0.13	0.05
+ 0.85	10.85	0.14	0.04
− 0.85	14.15	0.12	0.05
0.00	15.00	0.23	0.02
+ 0.85	15.85	0.20	0.05

The data emphasizes that the adaptive cutting method demonstrates higher accuracy and consistency in steel plate-beveling applications compared to the standard cutting method. This achievement is attributed to the integration of an ultrasonic thickness measurement sensor into a robotic plasma cutting system, enabling it to adjust and optimize cutting parameters and path planning based on material thickness adaptively and effectively. This finding aligns with the expectation that an adaptive approach can lead to more reliable and predictable outcomes, critical in industries where precision is paramount.

Overall, the ultrasonic-driven adaptive control of robotic plasma arc cutting for steel plate bevelling applications represents a smarter, more efficient approach to achieving precise and consistent bevel cuts across a wide spectrum of material thicknesses by automating the process, improving productivity, minimizing material waste, and enhancing cut quality. This adaptive approach is a significant departure from traditional plasma cutting approaches for weld bevel applications, which often relies on skilled operators for parameter settings and designing cutting process. Reducing the design time and enhancing cutting quality.

## Managerial insights and practical implications

- **Improved efficiency of workflow:**
  - Increases throughput and decreases production time by automating time-consuming operations.
  - Streamlines high-volume production.
- **Cost savings and optimizing resources**:
  - Minimizes material waste by accurate bevel cutting.
  - Minimize production costs by reducing labour costs.
- **Enhanced product quality**:
  - Delivers consistent and precise cuts, while adhering to standards.
  - Reduces defects, improves product reliability by lowering faults.
- **Workforce transformation**:
  - Transitions from manual cutting to setting up and supervision duties.
  - Creates safer working conditions and lower workplace hazards.

- **Integration with smart manufacturing**:
  - IoT-enabled systems offer real-time monitoring and predictive maintenance features.
  - Minimizes equipment downtime and guarantees process optimization.

- **Adaptability and scalability**:
  - Manages diverse bevel angle and root face requirements with ease.
  - Ideal for low-volume, high-mix production, allowing for quick adaptability.

- **Improved safety**:
  - Reduces exposure to hazards like fumes, sparks, and heat.
  - Reduces accidents and compliance with workplace safety standards.

- ** Environmental sustainability:**
  - Increases material utilization and energy efficiency.

## Conclusions

In recent years, the adoption of plasma arc cutting systems has significantly impacted heavy manufacturing industries such as oil and gas, nuclear, and shipbuilding. The exceptional efficiency and precision offered by plasma cutting systems have led to their widespread popularity. This article’s central focus lies in the automation of the metal plate bevelling process using a plasma cutting system integrated with automated real-time ultrasonic thickness measurement. The proposed approach addresses the challenges posed by manual and semi-automated processes, skill shortages, and quality control issues. The integration of a robotic manipulator, plasma cutting tool, and an ultrasonic sensor offers numerous advantages. By automating the plate bevelling process, manufacturers can achieve faster cycle times, higher output, and improved precision. The system’s ability to adapt to varying thicknesses, angles, and root faces without human intervention enhances flexibility and reduces errors. Additionally, this approach aligns with the Industry 4.0 vision, fostering increased competitiveness in the manufacturing sector.

The paper provides a detailed insight into the proposed framework, including the algorithms for automated bevel cutting planning, hardware design and output analysis.

- Analyzing the bevel angle deviations for both approaches. Standard cutting showed an observed range of 0.48 to 0.90 deg with material thickness tolerance varying from 7.15 to 15.85 mm, indicating increased deviation with greater thickness. Conversely, adaptive cutting exhibited a narrower range of 0.13 to 0.23 deg, suggesting consistent and lower bevel angle deviation compared to standard cutting.
- Analyzing the root face deviation. Standard cutting displayed deviations from 0.10 to 0.38 mm, with a tendency to increase as material thickness tolerance rises. A different pattern is observed in adaptive cutting, with deviations between 0.02 and 0.05 mm, maintaining significantly lower root face deviation, signifying enhanced cut accuracy regardless of material thickness tolerance.

The data underlines that the adaptive cutting approach showcased outstanding accuracy and consistency in metal plate bevel cut applications, attributed to integrating an ultrasonic thickness measurement sensor into the robotic plasma cutting system. This innovation enables adaptive adjustments of cutting parameters and path planning based on material thickness, resulting in improved control and precision of the cutting process. Such adaptability aligns with the expectation that an adaptive approach ensures more reliable outcomes, crucial in precision-driven industries.

### Limitations

The current system is limited to plane steel plate bevelling applications, which restricts its versatility in handling more complex three-dimensional bevelling applications.

### Future work

The future work will consider further research and experimentation into exploring the possibility of enhancing the process by integrating additional sensor modularity such as a laser scanner and camera system to reduce these deviations and improve overall efficiency.
